# David Hosack

**Published:** 1836-07

**Authors:** 


DR. HOSACK.

At New York, on the 22d December, 1835, David Hosack, M.D., F.R.S., L. and E., formerly Professor of Medicine in the University, and Physician to the Hospital in New York. Dr. Hosack was one of the most distinguished medical writers of America, and his works are well known in this country. The following are the titles of some of the principal:

Syllabus of Lectures on Botany, 8vo. New York, 1795.

Lecture on Medical Education, 8vo. New York, 1801.

Hortus Elgensis; or a Catalogue of Plants cultivated in the Elgin Botanic Garden, New York. New York, 1811.

A Statement of Facts relative to the Establishment and Progress of the Elgin Botanic Garden. New York, 1811.

Observations on the Laws governing Contagious Diseases. 4to. New York, 1815.

A System of Practical Nosology. First edition, 1818; second, 1821.

Essays on various Subjects of Medical Science. 8vo. 2 vols. 1824.

Observations on the Medical Character. 8vo. 1826.

Dr. Hosack died of apoplexy, occasioned, it is said by some of the foreign journals, by concern for the loss of 300,000 dollars in the great fire at New York. A German journal, in adverting to this circumstance, observes that few German doctors will die from a like cause. (Eine Todesursache, die unter den in Deutschland praktisirenden Aertzen nicht beobachtet sein soll!)

